# Effects of oat (*Avena sativa* L.) hay diet supplementation on the intestinal microbiome and metabolome of Small-tail Han sheep

**DOI:** 10.3389/fmicb.2022.1032622

**Published:** 2022-12-16

**Authors:** Shaofeng Su, Liwei Wang, Shaoyin Fu, Jie Zhao, Xiaolong He, Qiuju Chen, Damien P. Belobrajdic, Chuanzong Yu, Hongkui Liu, Haiqing Wu, Pingan Han, Bin Yang, Yao Huang, Yongbin Liu, Jiangfeng He

**Affiliations:** ^1^Inner Mongolia Academy of Agriculture and Husbandry Science, Hohhot, China; ^2^State Key Laboratory of Reproductive Regulation and Breeding of Grassland Livestock, Inner Mongolia University, Hohhot, China; ^3^Center of Reproductive Medicine, The Affiliated Hospital of Inner Mongolia Medical Hospital, Hohhot, China; ^4^Bayannur Institute of Agriculture and Animal Husbandry Science, Bayannur, China; ^5^CSIRO Health and Biosecurity, Adelaide, SA, Australia

**Keywords:** oat (*Avena sativa* L.), sheep, microbiome, metabolome, digestive tract, rumen

## Abstract

Supplementation of the sheep diet with oats (*Avena sativa* L.) improves animal growth and meat quality, however effects on intestinal microbes and their metabolites was not clear. This study aimed to establish the effect of dietary oat supplementation on rumen and colonic microbial abundance and explore the relationship with subsequent changes in digesta metabolites. Twenty Small-tail Han sheep were randomly assigned to a diet containing 30 g/100 g of maize straw (Control) or oat hay (Oat). After 90-days on experimental diets, rumen and colon digesta were collected and microbial diversity was determined by 16S rRNA gene Illumina NovaSeq sequencing and metabolomics was conducted using Ultra-high performance liquid chromatography Q-Exactive mass spectrometry (UHPLC-QE-MS). Compared to Control group, oat hay increased the abundance of Bacteroidetes and Fibrobacteres as well as known short-chain fatty acid (SCFA) producers Prevotellaceae, Ruminococcaceae and Fibrobacteraceae in rumen (*p* < 0.05). In rumen digesta, the Oat group showed had higher levels of (3Z,6Z)-3,6-nonadienal, Limonene-1,2-epoxide, P-tolualdehyde, and Salicylaldehyde compared to Control (*p* < 0.05) and these metabolites were positively correlated with the abundance of cecal *Prevotellaceae NK3B31*. In conclusion, supplementation of the sheep diet with oat hay improved desirable microbes and metabolites in the rumen, providing insight into mechanisms whereby meat quality can be improved by oat hay supplementation.

## Introduction

Oat is a high-quality feed crop for livestock, having strong adaptability, tillering ability, large biomass and high nutritional value (Andrzejewska et al., 2019). As an annual forage, oat has become the preferred feed for livestock and poultry breeding, while providing beneficial effects on growth performance and meat quality (Mwendia et al., 2018; Adewole et al., 2021; Donovan et al., 2021). Studies have shown that the digestibility of the dry matter and organic matter decreased with the increase of oat hay content in free-foraging mode (Long et al., 2004). Sontakke et al. (2019) found that adding oat hay to the diet significantly increased the fiber intake, reduced the emission of methane in buffalos. Diets containing oat hay improved the growth performance of calves, increased the apparent nutrient digestibility, and changed the rumen fermentation patterns (Zou et al., 2018; Gasiorek et al., 2020; Xiao et al., 2021). Meanwhile, lambs fed a combination diet containing oat hay produced the most satisfactory carcasses and chops (Whitney and Smith, 2015).

The microbes within the gastrointestinal tracts (GIT) play an important role in regulating metabolism of carbohydrates, amino acids, lipids, vitamins and minerals (Al-Lahham et al., 2010; Ussar et al., 2016) as well as the immune system of the host (Jia et al., 2008; Ley et al., 2008). Studies had shown that the change in animal feed directly affect the composition and abundance of GIT microbes (Morán et al., 2012). Dietary supplementation of oat hay can regulate rumen pH by changing the rumen microbial of calves (Lin et al., 2018). Feeding cattle oat hay in diet affected the microbiota, such that the Firmicutes/Bacteroidetes (F/B) ratio positively correlated with feed digestibility (Sim et al., 2022). Although studies have previously reported the ruman microbial changes following oat hay supplementation of sheep (Cui et al., 2019; An et al., 2020a), these findings were focused on microbial diversity in the rumen, and did not report changes in microbial metabolites or investigate different regions of GIT. Although the rumen is the primary site of fermentation and nutrient digestion in sheep, we were interested in understanding whether oat hay supplementation also effected microbe levels and production of metabolites in the distal region of GIT. These findings will help identify key metabolites for GIT health that are important for improved animal growth and meat quality.

## Materials and methods

### Animals

Twenty male Small-tailed Han sheep with similar genetic background (body weight of 16.8 ± 0.8 kg, around 90 days of age) were selected. They were randomly allocated to two groups. The diets contained 30 g/100 g Maize straw (Control) or 30 g/100 g oat hay (Oat) and the remainder of the diets containing similar ingredients as described in Supplementary Table S1. All sheep had free access to water during the trial. The experiment period lasted for 100 days, consisting of a 10-day preliminary adaptation period followed by a 90-day experimental feeding period. The ingredients and nutritional composition of the diets are presented in Supplementary Table S1. On the 100 th day, after 24 h without feed, sheep were slaughtered at a local slaughterhouse. This study was approved by the Animal Welfare Association and the Professional Committee of the Academy of Agricultural and Animal Husbandry Sciences of Inner Mongolia Autonomous Region (Hohhot, China; approval no. 2022003), in line with the Regulations on the Management of Experimental Animals.

### Sample collection

One day prior to slaughter, a veterinarian examined the sheep and confirmed that they had no gastrointestinal disease. After slaughter and dissection, all gastrointestinal organs were tied together with a rope between the narrow sections and placed horizontally to collect samples from the middle of each section. The sampling sites were consistent for each sheep. Digesta contents of stomach (in the rumen) and colon (in the middle of the ventral colon) were collected. Gastrointestinal digesta was handled and stored aseptically as possible to prevent contamination. The samples were stored in 50 mL sterile enzyme-free centrifuge tubes, immediately placed in liquid nitrogen and then stored in a freezer at −80°C.

### Microbial DNA extraction and sequencing

Total genomic DNA was extracted using DNeasy PowerSoil Kit (QIAGEN, Cat. No 12888) following the manufacturer’s instructions. Concentration of DNA was verified with NanoDrop and agarose gel. Genomic DNA was used as template for PCR amplification with the barcoded primers and Tks Gflex DNA Polymerase (Takara, Cat. No R060B). For bacterial diversity analysis, V3-V4 variable regions of 16S rRNA genes was amplified with universal primers 343 F (5′-TACGGRAGGCAGCAG-3′) and 798 R (5′-AGGGTATCTAATCCT-3′). After quality inspection and purification, another round of PCR amplification was performed. The final amplicon was quantified using Qubit dsDNA assay kit (Life Technologies, Cat. No Q32854). Equal amounts of purified amplicon were pooled for subsequent sequencing. The library was sequenced on an Illumina NovaSeq PE250 platform and 2 × 250 bp paired-end reads were generated. All library construction and sequencing was performed at the Oebiotech Company.

### Bioinformatics analysis

Raw sequencing data were in FASTQ format. Paired-end reads were then processed using Trimmomatic software (Bolger et al., 2014) to detect and cut off ambiguous bases (N). After trimming, paired-end reads were assembled using FLASH software (Reyon et al., 2012). Splicing Sequences were performed further denoising as follows: reads with ambiguous, homologous sequences or below 200 bp were abandoned. Reads with 75% of bases above Q20 were retained. Then, reads with chimera were detected and removed. These two steps were achieved using QIIME software (Caporaso et al., 2010; Version 1.8.0). Clean reads were subjected to primer sequences removal and clustering to generate operational taxonomic units (OTUs) using Vsearch software (Rognes et al., 2016) with 97% similarity cutoff. The representative read of each OTU was selected using QIIME package. All representative reads were annotated and blasted against Greengens database using RDP classifier (Wang et al., 2007; confidence threshold was 70%). The functional composition of the microorganisms was predicted by the PICRUSt (Version 1.1.2) programs.

### Metabolite extraction

After thawing at room temperature, 60 mg accurately weighed digesta sample was transferred to a 1.5 mL EP tube (Axygen, Cat. No MCT-150-C). 20 µL internal standard (2-chloro-l-phenylalanine in methanol, 0.3 mg/mL) and 600 µL extraction solvent with methanol/water (4/1, *v/v*) were added to each tube. Two small steel balls (2 mm) were added to the tube. Samples were stored at −20°C for 5 min and then homogenized at 60 Hz for 2 min, ultrasonic extraction in ice water bath for 10 min, stored at −20°C for 30 min. The tubes were then centrifuged at 12,000× *g*, 4°C for 10 min. 300 µL of supernatant was removed from each tube and placed in a brown glass vial and then dried in a freeze concentration centrifugal dryer (Zuoyan technology, Shanghai, China). 400 µL mixture of methanol and water (1/4, *v/v*) were added to each sample, samples vortexed for 30 s, sonicated for 3 min (4°C water bath) and then stored at −20°C for 2 h. Samples were centrifuged at 12,000× *g*, 4°C for 10 min. The supernatant (150 µL) from each tube were collected using crystal syringes, filtered through 0.22 μm microfilters and transferred to LC/MS glass vial. The vials were stored at −80°C until anylsed by LC–MS. Quality control (QC) samples were prepared by mixing an equal volume of all of the extracts together and 150 µL of the supernatant was used for the UHPLC-QE-MS analysis.

### LC/MS analysis

The LC/MS analyzes were performed by UHPLC-QE-MS (Thermo Fisher Scientific, Waltham, MA, USA) with a heated electrospray ionization (ESI) source (Thermo Fisher Scientific, Waltham, MA, USA). This was used to analyze the metabolic profiling in both ESI positive and ESI negative ion modes. An ACQUITY UPLC HSS T3 (100 mm × 2.1 mm, 1.8 μm, Waters) coupled to QE plus (Thermo Fisher Scientific) were employed. The binary gradient elution system consisted of (A) water (containing 0.1% formic acid, *v/v*) and (B) acetonitrile (containing 0.1% formic acid, *v/v*) and separation was achieved using the following gradient: 0–2 min, 5% B; 4 min, 25% B; 8 min, 50% B; 10 min, 80% B; 14–15 min, 100% B and 15.1–16 min, 5% B, which was delivered at 0.35 mL/min and column temperature was 45°C. All samples were kept at 4°C during the analysis. The injection volume was 2 µL. The mass range was from *m/z* 100 to 1,000. The resolution was set at 70,000 for the full MS scans and 17,500 for HCD MS/MS scans. The collision energy was set at 10, 20 and 40 eV. The mass spectrometer operated as follows: spray voltage, 3,800 V (+) and 3,000 V (−); sheath gas flow rate, 35 arbitrary units; auxiliary gas flow rate, 8 arbitrary units; capillary temperature, 320°C. The QCs were injected at regular intervals (every 10 samples) throughout the analytical run to provide a set of data from which repeatability can be assessed.

### Metabolomic analysis

The non-targeted metabolites were analyzed by liquid chromatography-mass spectrometry (LC–MS) from Shanghai Luming Biological Technology Co., Ltd. The data was pretreated using Progenesis QI v2.3 software (Nonlinear Dynamics, Newcastle, UK). Multivariate analyzes (principal component analysis, PCA; and orthogonal projections to latent structures discriminant analysis, OPLS-DA) were used to find out the differential metabolites between groups (Nicholson et al., 1999; Trygg and Wold, 2002). Univariate statistical analysis was used to identify the difference in metabolites between the groups. The enriched pathway analysis of differential metabolites was performed using KEGG database^1^ and R program (Version 3.4.1).

### Statistical analysis

SPSS 20.0 software (IBM SPSS, Chicago, IL) was used for statistical analysis, and the data was reported as mean ± SD, with significance reported at a level of *p* < 0.05. Statistical significance was analyzed by ANOVA, and multiple groups were compared using the LSD test. Spearman correlation was used to calculate the correlation between the relative abundance of microorganisms (Genus) and the response intensity data of corresponding metabolites (Kong et al., 2013; Schwab et al., 2014).

## Results

### Digesta microbial abundance and diversity

**Figure 1:**
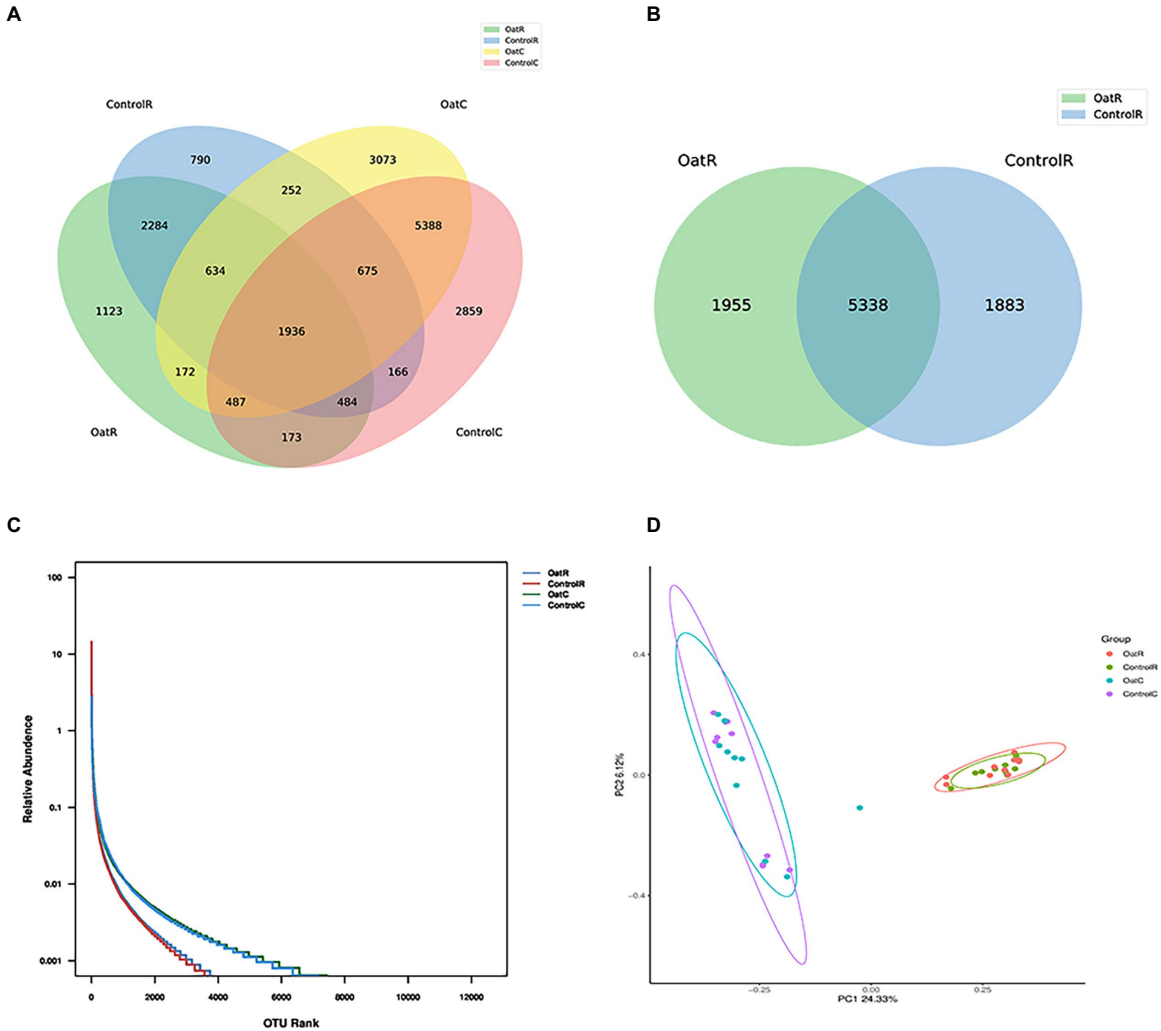
Effect of oat hay on the microbial richness of the Small-tail Han sheep rumen digesta. **(A)** Venn diagram of OTUs in the Small-tail Han sheep GIT microbiota. **(B)** Venn diagram of OTUs in the Small-tail Han sheep rumen microbiota. **(C)** Rank abundance rarefaction curve of GIT contents of Small-tail Han sheep. **(D)** Principal component analysis (PCA) with clustering representing the dissimilarity of bacterial structure found among samples from Small-tail Han sheep GIT compartments. OatR, Rumen samples from oat group; OatC, Colon samples from oat group; ControlR, Rumen samples from control group; ControlC, Colon samples from control group.

The data of clean tags obtained by removing chimerism (that is, the data finally used for analysis) was distributed between 46,861 and 72,105. The number of OTU of each sample was distributed between 1,556 and 4,071 (Supplementary Table S2). For the two regions of digesta analyzed (rumen and colon) and the two dietary treatments, cluster analysis showed that there were 1,936 shared OTUs. Of these, 26% of OTUs were similar across rumen digesta samples for Oat and Control, whereas 5.5% of OTUs were unique to the rumen digesta of Oat fed sheep and 3.9% were unique to the rumen digesta of Control fed sheep (Figures 1A,B). For colon digesta, 41% of OTUs were similar to Oat and Control, whereas 15% of OTUs were unique to the colonic digesta of Control fed sheep and 14% were unique to Oat fed sheep. However, there was no significant difference in the number of different OTUs between colonic digesta samples from Oat and Control groups (Figure 1A; Supplementary Figure S1).

Each exponential dilution curve showed that the sequencing depth of all samples covered the GIT microbiome (Supplementary Figure S2). The four groups could be divided into two distinct regions, with the colon digesta having greater abundance of microbes compared to the rumen digesta (Figure 1C). The richness and diversity index of microbes in rumen and colon digesta are shown in Table 1. The good coverage index was between 0.98 and 0.99, indicating that more than 98% of the bacterial groups were present in all samples analyzed, which better reflected the bacterial community of samples. Colon digesta compared to rumen digesta, had a higher species richness index (observed Species and Chao1) and higher species diversity index (PD whole tree, Shannon and Simpson index; *p* < 0.01, Table 1). In addition, the colonic digesta samples also showed greater scattering of data points in PCoA analysis, which suggests larger differences in microbial communities compared to rumen digesta (Figure 1D).

**Table 1:** Effect of oat hay on the microbial diversity in rumen and colonic digesta of Small-tail Han sheep.

Sample name	PD whole tree	Chao1	Goods coverage	Observed species	Shannon	Simpson
Rumen digesta Oat	71.6 ± 14.4^B^	2787.85 ± 642.57^B^	0.98 ± 0.004^B^	1918.58 ± 500.98^B^	6.75 ± 0.58^B^	0.951 ± 0.022^B^
Control	73.6 ± 12.0^B^	2944.86 ± 460.60^B^	0.98 ± 0.003^B^	1967.26 ± 411.82^B^	6.59 ± 0.65^B^	0.941 ± 0.032^B^
Colon digesta Oat	110.5 ± 21.3^A^	4096.22 ± 876.22^A^	0.98 ± 0.005^A^	2945.23 ± 711.35^A^	8.09 ± 0.91^A^	0.978 ± 0.012^A^
Control	111.9 ± 24.8^A^	4223.05 ± 622.86^A^	0.98 ± 0.004^A^	2988.76 ± 503.13^A^	8.20 ± 0.82^A^	0.979 ± 0.019^A^

(1) Analysis of different samples under the threshold of 97% identity. (2) Different uppercase letters means the significance at *p* < 0.01 and same letters in the superscripts represent *p* > 0.05.

### Effect of oat on rumen and colonic digesta microbial abundance

**Figure 2:**
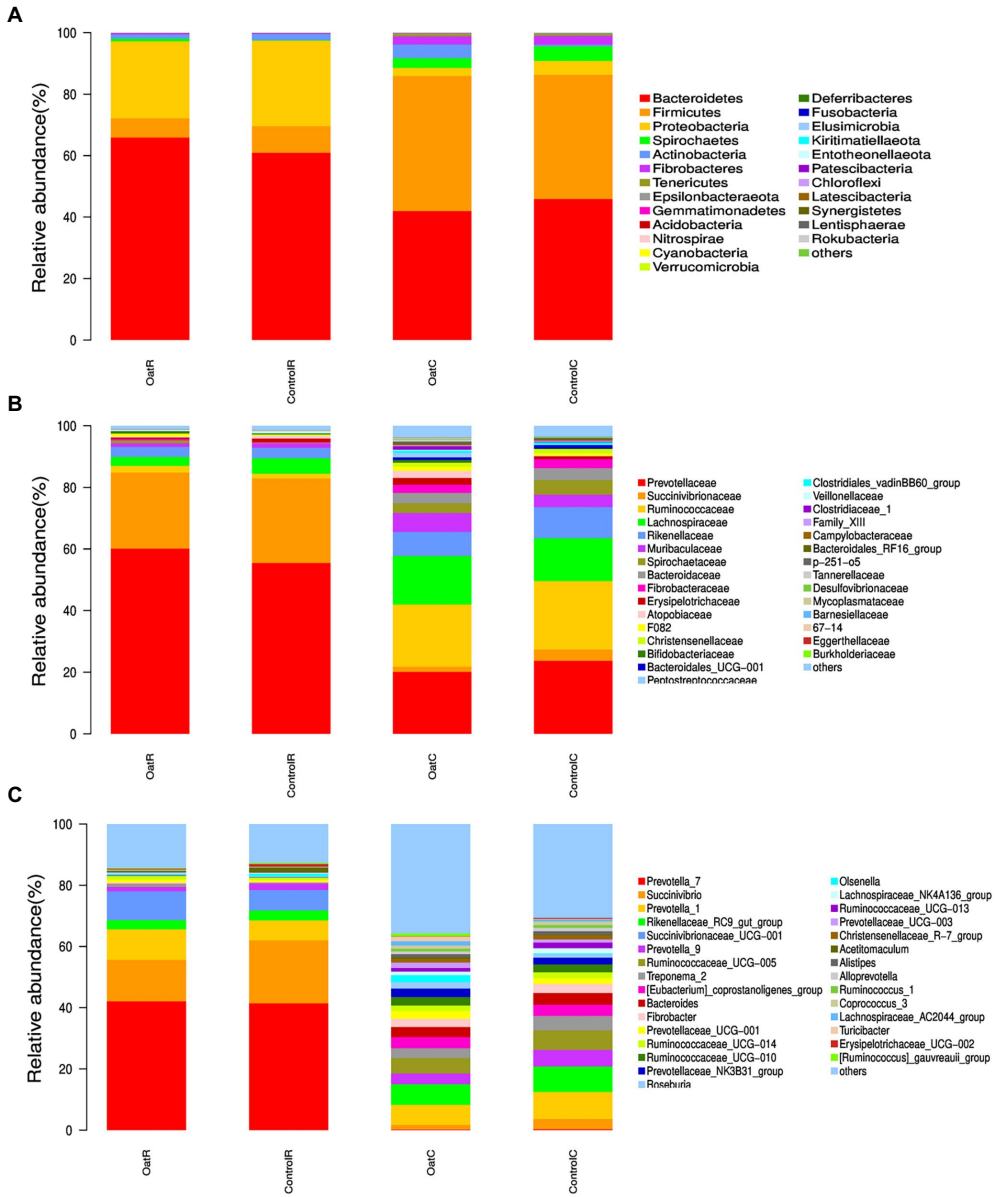
Relative microbial abundance of rumen and colonic digesta from Small-tail Han sheep. Phylum **(A)**, Family **(B)**, and Genus levels **(C)**. OatR, Rumen samples from oat group; OatC, Colon samples from oat group; ControlR, Rumen samples from control group; ControlC, Colon samples from control group.

At phylum level, there were 6 species with relative abundance greater than 1% of the microflora in rumen and colonic digesta (OatR *vs.* ControlR, OatC *vs.* ControlC) of Small-tail Han sheep, including Bacteroidetes (65.9% *vs.* 60.9%, 42.0% *vs.* 45.9%), Proteobacteria (25.0% *vs.* 27.8%, 2.6% *vs.* 4.5%), Firmicutes (6.3% *vs.* 8.6%, 43.9% *vs.* 40.4%), Spirochaetes (0.9% *vs.* 0.2%, 3.2% *vs.* 4.7%), Fibrobacteres (0.44% *vs.* 0.3%, 2.7% *vs.* 2.9%) and Actinobacteria (1.4% *vs.* 2.0%, 4.4% *vs.* 0.5%; Figure 2A). The Oat group had a higher relative abundance of Bacteroidetes and Fibrobacteres in rumen digesta compared to Control (*p* < 0.05). However, microbial abundance in colonic digesta was similar for Oat and Control (Supplementary Figure S3).

In the rumen digesta of Oat fed sheep the relative abundance of Prevotellaceae (60.14% *vs.* 55.45%), Ruminococcaceae (2.18% *vs.* 1.51%) and Fibrobacteraceae (0.44% *vs.* 0.28%) was higher than the Control fed sheep (*p* < 0.05; Figure 2B; Supplementary Figure S4). At the genus level, compared to the Control group, the Oat group had significantly higher relative abundance of *Prevotellaceae NK3B31 group* (0.13% *vs.* 0.08%), *Eubacterium xylanophilum group*, *Anaerovorax, Anaerotruncus* and *Enterorhabdus* in rumen digesta (*p* < 0.05), whereas *Sharpea, LachnospiraceAE UCG-001* and *Desulfobulbus* decreased in the OatR group (*p* < 0.05; Figure 2C; Supplementary Figure S5).

The metabolic function of the microbiota were predicted using PICRUSt and KEGG (Kyoto Encyclopedia of Genes and Genomes) databases. At the second level, there were 42 predicted functions of the rumen group with significant differences, among which the functions of the Digestive System and Excretory System of the Oat which were present at higher levels compared with Control (Supplementary Figure S6).

### Effect of oat on rumen and colonic digesta metabolites

The unsupervised PCA plot showed separation for rumen digesta metabolites between Oat and Control (Supplementary Figure S7A). As indicated by the OPLS-DA score, the metabolites of the rumen group were well separated (Supplementary Figures S7B,C). The parameters for the classification from the software were stable and relevant to fitness and prediction (Supplementary Table S3).

**Figure 3:**
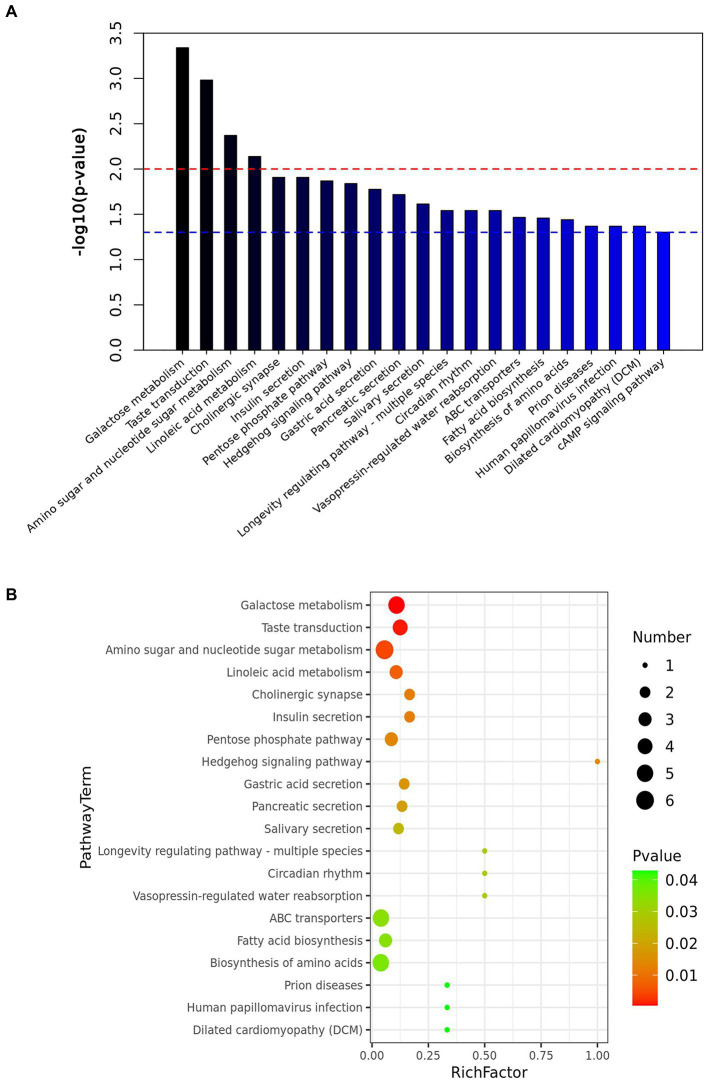
Effect of dietary oat hay on the metabolites in the rumen digesta of Small-tail Han sheep. **(A)** Enrichment diagram of metabolic pathways; **(B)** Bubble map of metabolic pathways. OatR vs. ControlR, OatR, Rumen samples from oat group; ControlR, Rumen samples from control group.

OPLS-DA analysis was used to screen the differential metabolites between groups. The screening criteria were VIP value of OPLS-DA model >1 and *p*-value of *T*-test <0.05. A total of 558 differential metabolites were identified in the rumen digesta of Oat and Control, among which 328 were up-regulated and 230 were down-regulated (Supplementary Figure S7D). The enrichment pathways with significant differences included galactose metabolism, amino sugar and nucleotide sugar metabolism, linoleic acid metabolism, pentose phosphate pathway, gastric acid secretion, fatty acid biosynthesis and biosynthesis of amino acids (*p* < 0.05), and these pathways play important roles in the regulation of animal growth and development (Figure 3A).

### Correlations between microbial communities and metabolites

In rumen digesta, a number of metabolites showed that (Table 2; Supplementary Figure S8), (3Z,6Z)-3,6-nonadienal, Limonene-1,2-epoxide, P-tolualdehyde, and Salicylaldehyde were positively correlated with *Prevotellacea NK3B31 group* (*p* < 0.05), and negatively correlated with *Desulfobulbus* (*p* < 0.01); D-galactose was positively correlated with *Desulfobulbus* (*p* < 0.05) and negatively correlated with *Prevotellaceae NK3B31 group* (*p* < 0.05). The sucrose, Beta-lactose and D-maltose were positively correlated with *Lachnospiraceae UCG-010* (*p* < 0.05), and negatively correlated with *Prevotellaceae NK3B31 group* (*p* < 0.01). These metabolites are mainly enriched by amino sugar and nucleotide sugar metabolism, biosynthesis of amino acids, and galactose metabolism (Figure 3B).

**Table 2:** Differences in candidate rumen or colon metabolites between OatR and ControlR.

Compounds	VIP	*P*-value	Log2(FC)
Acetylcholine	3.6374	0.0370	0.6291
(3Z,6Z)-3,6-nonadienal	3.4675	0.0009	0.3466
Limonene-1,2-epoxide	6.1414	0.0018	0.3303
P-tolualdehyde	3.3897	0.0018	0.3266
Cyclopentanone	3.8708	0.0109	0.3151
Phosphohydroxypyruvic acid	3.3308	0.0059	0.3145
Salicylaldehyde	2.5428	0.0273	0.1905
D-galactose	4.8168	0.0305	−1.3473
Sucrose	15.7878	0.0353	−2.4083
Beta-lactose	10.0234	0.0332	−2.4354
D-maltose	7.1842	0.0279	−3.0623

The screening criteria were VIP >1 and *p* < 0.05 of t-test. VIP (Variable important in projection): The VIP value from the OPLS-DA model, the larger the VIP, the greater the contribution of this variable to the grouping. Log2 (FC): The ratio of the mean expression levels of metabolites in the two groups of samples, with positive values indicating up-regulation and negative values indicating down-regulation.

## Discussion

Oat hay has been widely used in the sheep diet as it provides greater acid detergent fiber (ADF) and neutral detergent fiber (NDF), which contributes to maintaining the homeostasis of rumen environment and the growth of microorganisms (Wang et al., 2016). In the current study, colon microbial diversity was similar for the oat and control fed sheep. Although overall diversity is not affected by oat supplementation, there were changes in the current study there were specific changes in rumen microbes. We showed that when sheep were fed oats they had higher abundance of Bacteroidetes and Fibrobacteres in the rumen compared to those fed Maize straw (*p* < 0.05). These changes are consistent with previous studies which showed that the abundance of Fibrobacteres, Ruminococcaceae and Prevotella in the rumen of sheep change in response to the digestibility of feed provided (Cui et al., 2019; An et al., 2020a). Supplementation of the sheep diet with oat hay was also shown to increase the abundance of Bacteroidete, a dominant bacteria species in the rumen digesta (An et al., 2020a). The increase of Bacteroidetes and Firmicutes can effectively improve the digestibility of dietary fiber (Xu et al., 2015). Thus, the higher abundance of Bacteroidetes and Firmicutes in the rumen digesta of Small-tail Han sheep suggest greater digestion of the fibre in the diet. Fibrobacteres can decompose cellulose and hemicellulose in feed, produce acetic acid, propionic acid and butyric acid, and provide energy for host through the tricarboxylic acid cycle. There are a large number of Bacteroidetes and Fibrobacetes in the rumen of Small-tail Han sheep, which indicates that they are more suitable for vegetative food resources, and it also proves once again that sheep are rumen fermenting animals. Studies have shown that in the rumen of cattle fed forages high in neutral detergent fiber, the abundance of Fibrobacteres was significantly increased by oat hay, and cellulose components significantly affected microbial colonization and ultimately affected the digestion of herbage (Gharechahi et al., 2020). Therefore, dietary oat supplementation may improve the degradation rate of fiber components in the rumen, and produce more short-chain fatty acids, leading to better rumen fermentation effect. In the prediction of microbial function, Oat was also significantly enriched in the functions of Digestive System and Excretory System of Control, which showed that oat could increase the abundance of plant decomposition bacteria in the rumen. Although the rumen is the primary site of GIT fermentation, we were interested in exploring how oat supplementation effects microbial abundance in the colon. At present, there are few reports about the effects of oat hay on colon microbe of ruminants. The results of this experiment show that OatC and ControlC had similar bacterial abundance, suggesting that oat mainly altered ruminal bacterial community structure with little effect on colonic microbes.

At the family level, OatR group significantly increased the relative abundance of Prevotellaceae Ruminococcaceae and Fibrobacteraceae compared with ControlR group (*p* < 0.05; Figure 2B; Supplementary Figure S4). Prevotellaceae, a strain of Prevotella with enzymes that can degrade cellulose and xylan, is also considered a beneficial bacterium. At the same time, Prevotellaceae can produce propionate through sugar or lactic acid fermentation. Therefore, Prevotellaceae is considered to be related to the synthesis of short-chain fatty acids (SCFAs; Shen et al., 2017; Song et al., 2019; Attaye et al., 2022). Ruminococcaceae is rich in hydrolases, which mainly act on carbohydrates and have higher specificity for the degradation of complex macromolecules in feed (Ozbayram et al., 2018). Fibrobacteraceae can significantly improve the degradation of cellulose in feed, thus affecting the final digestion level of forage (Gharechahi et al., 2020). At the genus level, the OatR group significantly improved *Prevotellaceae NK3B31 group* and *Eubacterium Xylanophilum* for ControlR group relative abundance, *Anaerovorax*, *Anaerotruncus* and *Enterorhabdus* (*p* < 0.05), Sharpea, *LachnospiraceAE UCG-001* and *Desulfobulbus* have decreased significantly (*p* < 0.05; Figure 2C; Supplementary Figure S5). Studies have shown that *Prevotella* is abundant in the digestive tracts of individuals fed a carbohydrate and fiber diet, which is consistent with the results of oat feeding in this study (Jiang et al., 2020).

In the oat group, *Prevotellaceae NK3B31 group* was positively correlated with rumen metabolites (3Z,6Z)-3,6-nonadienal, Limonene-1,2-epoxide, P-tolualdehyde and Salicylaldehyde. Sucrose is negatively related to D-galactose, Sucrose, beta-lactose and D-maltose. These metabolite synthesis pathways is known to be involved in glucose metabolism. *Prevotellaceae NK3B31 group* play an important role in the synthesis of these metabolites. (3Z,6Z)-3,6-nonadienal is widely used in the production of edible flavors (An et al., 2020b). Limonene-1,2-epoxide is an oxidized product of Limonene. Limonene is a natural and effective anti-injury and anti-tumor compound, widely existing in monocyclic terpenes in natural plant essential oils (Sun, 2007). Limonene-1,2-epoxide is a very useful intermediate in organic synthesis, which can obtain a series of important organic compounds through ring-opening reaction with a variety of nucleophiles, and these compounds are widely used in medicine and flavors (Souto et al., 2020). P-tolualdehyde and Salicylaldehyde are also important spice components and are widely used as intermediates in organic synthesis (Realini et al., 2017; Api et al., 2021). Oat increases the content of these metabolites in rumen digesta, while the addition of certain metabolites (such as Limonene, Nonadienal) to feed can improve the meat texture by increasing the content of total volatile fatty acids in rumen and changing the composition of fatty acids, increasing the consumer’s preference and its economic return (Jiang and Xiong, 2016; Realini et al., 2017; Van Houcke et al., 2017; Temmar et al., 2021)*. Prevotellaceae NK3B31 group* was negatively correlated with carbohydrate metabolites, indicating that the genus may decompose and consume carbohydrate substances in large quantities, because *Prevotellaceae NK3B31 group* may play an important role in the synthesis of these metabolites through sugar metabolism pathway. *Lachnospiraceae UCG-010* was positively correlated with carbohydrate metabolites. In the rumen of Oat group, the relative abundance of *Lachnospiraceae UCG-010* was significantly decreased. In related studies of diabetes, *Lachnospiraceae UCG-010* was found to be closely related to elevated blood glucose level (*p* < 0.01; Zhang et al., 2017; Yang et al., 2021).

## Conclusion

This study provides further evidence that oat hay supplementation in the sheep diet increases the abundance of rumen bacteria, such as Bacteroidetes and Fibrobacteres. At the family level, oat hay significantly increased the abundance of Prevotellaceae, Ruminococcaceae and Fibrobacteraceae associated with the synthesis of SCFAs. Furthermore, it increased the content of (3Z,6Z)-3,6-nonadienal, Limonene-1,2-epoxide, P-tolualdehyde, and Salicylaldehyde in the rumen digesta which is associated with improving meat quality. The above metabolin substances were positively correlated with the *Prevotellaceae NK3B31 group*. Oat hay had little effect on colon microbial diversity. In summary, this study provides a deeper understanding how oat supplementation may regulate metabolism and improve performance of sheep through stimulating the metabolic activity of specific microbes.

## Data availability statement

The data presented in the study are deposited in the NCBI repository, accession number PRJNA878668. The original data of this study are included in the article or Supplementary Material. Further inquiries can be directed to the corresponding authors.

## Ethics statement

The animal study was reviewed and approved by the animal study was reviewed and approved by the Guidelines of the Institutional Animal Care and Use Committee of the Biological Technology Research Institute, Inner Mongolia Academy of Agriculture and Husbandry Science. Written informed consent was obtained from the owners for the participation of their animals in this study.

## Author contributions

SS and LW: conceptualization, data analysis, and drafting the manuscript. SF, JZ, and XH: animal feeding, sampling, and determination. CY and QC: diet formulation. DB, HW, BY, and YH: supervision, review, and editing. HL: final approval of the manuscript. YL and JH: conception, design and financial support. All authors contributed to the article and approved the final manuscript.

## Funding

This work was supported by the Major Science and Technology Projects of the Inner Mongolia Autonomous Region (2020ZD0003), Inner Mongolia Autonomous Region Science and Technology Plan Project (2021GG0030 and 2021GG0012), the “Science and Technology to Revitalize Mongolia” Key Project of Inner Mongolia Autonomous Region (NMKJXM202110), National technical system of mutton sheep production (CARS-38), Innovation Fund of Inner Mongolia Academy of Agricultural and Animal Husbandry Science (2020CXYJJN13 and 2022CXJJM04), and Talent Introduction Project of State Administration of Foreign Experts Affairs (G2022005001), High End Foreign Expert Program (G2021005002L).

## Conflict of interest

The authors declare that the research was conducted in the absence of any commercial or financial relationships that could be construed as a potential conflict of interest.

## Publisher’s note

All claims expressed in this article are solely those of the authors and do not necessarily represent those of their affiliated organizations, or those of the publisher, the editors and the reviewers. Any product that may be evaluated in this article, or claim that may be made by its manufacturer, is not guaranteed or endorsed by the publisher.

## Footnotes

^1^ http://www.genome.jp/KEGG/pathway.html

## References

[ref1] Adewole D. I. Oladokun S. Santin E. (2021). Effect of organic acids-essential oils blend and oat fiber combination on broiler chicken growth performance, blood parameters, and intestinal health. Anim. Nutr. 7, 1039–1051. doi: 10.1016/j.aninu.2021.02.001, PMID: 34738034 PMC8546314

[ref2] Al-Lahham S. H. Roelofsen H. Priebe M. Weening D. Dijkstra M. Hoek A.. (2010). Regulation of adipokine production in human adipose tissue by propionic acid. Eur. J. Clin. Investig. 40, 401–407. doi: 10.1111/j.1365-2362.2010.02278.x, PMID: 20353437

[ref3] An Y. Qian Y. L. Alcazar Magana A. Xiong S. Qian M. C. (2020b). Comparative characterization of aroma compounds in silver carp (Hypophthalmichthys molitrix), pacific whiting (Merluccius productus), and Alaska Pollock (Theragra chalcogramma) surimi by aroma extract dilution analysis, odor activity value, and aroma recombination studies. J. Agric. Food Chem. 68, 10403–10413. doi: 10.1021/acs.jafc.9b07621, PMID: 32146815

[ref4] An X. Zhang L. Luo J. Zhao S. Jiao T. (2020a). Effects of oat Hay content in diets on nutrient metabolism and the rumen microflora in sheep. Animals 10:2341. doi: 10.3390/ani10122341, PMID: 33317030 PMC7763615

[ref5] Andrzejewska J. Contreras-Govea F. E. Pastuszka A. Kotwica K. Albrecht K. A. (2019). Performance of oat (*Avena sativa* L.) sown in late summer for autumn forage production in Central Europe. Grass Forage Sci. 74, 97–103. doi: 10.1111/gfs.12400

[ref6] Api A. Belsito D. Biserta S. Botelho D. Bruze M. Burton G. Jr.. (2021). RIFM fragrance ingredient safety assessment, p-tolualdehyde, CAS registry number 104-87-0. Food Chem. Toxicol. 149:111982. doi: 10.1016/j.fct.2021.111982, PMID: 33454360

[ref7] Attaye I. Warmbrunn M. V. Boot A. N. van der Wolk S. C. Hutten B. A. Daams J. G.. (2022). A systematic review and meta-analysis of dietary interventions modulating gut microbiota and cardiometabolic diseases–striving for new standards in microbiome studies. Gastroenterology 162, 1911–1932. doi: 10.1053/j.gastro.2022.02.011, PMID: 35151697

[ref8] Bolger A. M. Lohse M. Usadel B. (2014). Trimmomatic: a flexible trimmer for Illumina sequence data. Bioinformatics 30, 2114–2120. doi: 10.1093/bioinformatics/btu170, PMID: 24695404 PMC4103590

[ref9] Caporaso J. G. Kuczynski J. Stombaugh J. Bittinger K. Bushman F. D. Costello E. K.. (2010). QIIME allows analysis of high-throughput community sequencing data. Nat. Methods 7, 335–336. doi: 10.1038/nmeth.f.303, PMID: 20383131 PMC3156573

[ref10] Cui X. Wang Z. Yan T. Chang S. Wang H. Hou F. (2019). Rumen bacterial diversity of Tibetan sheep (*Ovis aries*) associated with different forage types on the Qinghai-Tibetan Plateau. Can. J. Microbiol. 65, 859–869. doi: 10.1139/cjm-2019-0154, PMID: 31386822

[ref11] Donovan B. Suarez-Trujillo A. Casey T. Aryal U. K. Conklin D. Williams L. L.. (2021). Inclusion of oat and yeast culture in sow gestational and lactational diets alters immune and antimicrobial associated proteins in Milk. Animals 11:497. doi: 10.3390/ani11020497, PMID: 33672799 PMC7918739

[ref12] Gasiorek M. Stefanska B. Pruszynska-Oszmalek E. Taciak M. Komisarek J. Nowak W. (2020). Effect of oat hay provision method on growth performance, rumen fermentation and blood metabolites of dairy calves during preweaning and postweaning periods. Animal 14, 2054–2062. doi: 10.1017/S1751731120000774, PMID: 32308189

[ref13] Gharechahi J. Vahidi M. F. Ding X.-Z. Han J.-L. Salekdeh G. H. (2020). Temporal changes in microbial communities attached to forages with different lignocellulosic compositions in cattle rumen. FEMS Microbiol. Ecol. 96:fiaa069. doi: 10.1093/femsec/fiaa069, PMID: 32304321

[ref14] Jia W. Li H. Zhao L. Nicholson J. K. (2008). Gut microbiota: a potential new territory for drug targeting. Nat. Rev. Drug Discov. 7, 123–129. doi: 10.1038/nrd2505, PMID: 18239669

[ref15] Jiang X. Lu N. Zhao H. Yuan H. Xia D. Lei H. (2020). The microbiome–metabolome response in the colon of piglets under the status of weaning stress. Front. Microbiol. 11:2055. doi: 10.3389/fmicb.2020.02055, PMID: 32983040 PMC7483555

[ref16] Jiang J. Xiong Y. L. (2016). Natural antioxidants as food and feed additives to promote health benefits and quality of meat products: a review. Meat Sci. 120, 107–117. doi: 10.1016/j.meatsci.2016.04.005, PMID: 27091079

[ref17] Kong L.-C. Tap J. Aron-Wisnewsky J. Pelloux V. Basdevant A. Bouillot J.-L.. (2013). Gut microbiota after gastric bypass in human obesity: increased richness and associations of bacterial genera with adipose tissue genes. Am. J. Clin. Nutr. 98, 16–24. doi: 10.3945/ajcn.113.058743, PMID: 23719559

[ref18] Ley R. E. Hamady M. Lozupone C. Turnbaugh P. J. Ramey R. R. Bircher J. S.. (2008). Evolution of mammals and their gut microbes. Science 320, 1647–1651. doi: 10.1126/science.1155725, PMID: 18497261 PMC2649005

[ref19] Lin X. Wang J. Hou Q. Wang Y. Hu Z. Shi K.. (2018). Effect of hay supplementation timing on rumen microbiota in suckling calves. Microbiol. Open 7:e00430. doi: 10.1002/mbo3.430, PMID: 29280327 PMC5822350

[ref20] Long R. Dong S. Hu Z. Shi J. Dong Q. Han X. (2004). Digestibility, nutrient balance and urinary purine derivative excretion in dry yak cows fed oat hay at different levels of intake. Livest. Prod. Sci. 88, 27–32. doi: 10.1016/j.livprodsci.2003.11.004

[ref21] Morán L. Andrés S. Bodas R. Benavides J. Prieto N. Pérez V.. (2012). Antioxidants included in the diet of fattening lambs: effects on immune response, stress, welfare and distal gut microbiota. Anim. Feed Sci. Technol. 173, 177–185. doi: 10.1016/j.anifeedsci.2012.01.010

[ref22] Mwendia S. W. Mwungu C. M. Ng’ang’a S. K. Njenga D. Notenbaert A. (2018). Effect of feeding oat and vetch forages on milk production and quality in smallholder dairy farms in Central Kenya. Trop. Anim. Health Prod. 50, 1051–1057. doi: 10.1007/s11250-018-1529-3, PMID: 29427246

[ref23] Nicholson J. K. Lindon J. C. Holmes E. (1999). 'Metabonomics': understanding the metabolic responses of living systems to pathophysiological stimuli via multivariate statistical analysis of biological NMR spectroscopic data. Xenobiotica 29, 1181–1189. doi: 10.1080/004982599238047, PMID: 10598751

[ref24] Ozbayram E. G. Ince O. Ince B. Harms H. Kleinsteuber S. (2018). Comparison of rumen and manure microbiomes and implications for the inoculation of anaerobic digesters. Microorganisms 6:15. doi: 10.3390/microorganisms6010015, PMID: 29443879 PMC5874629

[ref25] Realini C. Bianchi G. Bentancur O. Garibotto G. (2017). Effect of supplementation with linseed or a blend of aromatic spices and time on feed on fatty acid composition, meat quality and consumer liking of meat from lambs fed dehydrated alfalfa or corn. Meat Sci. 127, 21–29. doi: 10.1016/j.meatsci.2016.12.013 28110126

[ref26] Reyon D. Tsai S. Q. Khayter C. Foden J. A. Sander J. D. Joung J. K. (2012). FLASH assembly of TALENs for high-throughput genome editing. Nat. Biotechnol. 30, 460–465. doi: 10.1038/nbt.2170, PMID: 22484455 PMC3558947

[ref27] Rognes T. Flouri T. Nichols B. Quince C. Mahé F. (2016). VSEARCH: a versatile open source tool for metagenomics. Peer J. 4:e2584. doi: 10.7717/peerj.2584, PMID: 27781170 PMC5075697

[ref28] Schwab C. Berry D. Rauch I. Rennisch I. Ramesmayer J. Hainzl E.. (2014). Longitudinal study of murine microbiota activity and interactions with the host during acute inflammation and recovery. ISME J. 8, 1101–1114. doi: 10.1038/ismej.2013.223, PMID: 24401855 PMC3996699

[ref29] Shen F. Zheng R.-D. Sun X.-Q. Ding W.-J. Wang X.-Y. Fan J.-G. (2017). Gut microbiota dysbiosis in patients with non-alcoholic fatty liver disease. Hepatobiliary Pancreat. Dis. Int. 16, 375–381. doi: 10.1016/S1499-3872(17)60019-5 28823367

[ref30] Sim S. Lee H. Yoon S. Seon H. Park C. Kim M. (2022). The impact of different diets and genders on fecal microbiota in Hanwoo cattle. J. Anim. Sci. Technol. 64, 897–910. doi: 10.5187/jast.2022.e71, PMID: 36287745 PMC9574620

[ref31] Song X. Zhong L. Lyu N. Liu F. Li B. Hao Y.. (2019). Inulin can alleviate metabolism disorders in Ob/Ob mice by partially restoring leptin-related pathways mediated by gut microbiota. Genom. Proteom. Bioinform. 17, 64–75. doi: 10.1016/j.gpb.2019.03.001, PMID: 31026583 PMC6520907

[ref32] Sontakke U. Prusty S. Kundu S. Sharma V. K. (2019). Comparative evaluation of oat hay and silage based rations on nutrient utilization and methane emissions in murrah buffaloes. Indian J. Anim. Nutr. 36, 347–352. doi: 10.5958/2231-6744.2019.00057.4

[ref33] Souto E. B. Zielinska A. Souto S. B. Durazzo A. Lucarini M. Santini A.. (2020). (+)-limonene 1, 2-epoxide-loaded slns: evaluation of drug release, antioxidant activity, and cytotoxicity in an HaCaT cell line. Int. J. Mol. Sci. 21:1449. doi: 10.3390/ijms21041449, PMID: 32093358 PMC7073088

[ref34] Sun J. (2007). D-limonene: safety and clinical applications. Altern. Med. Rev. 12, 259–264. PMID: 18072821

[ref35] Temmar R. Rodríguez-Prado M. Forgeard G. Rougier C. Calsamiglia S. (2021). Interactions among natural active ingredients to improve the efficiency of rumen fermentation in vitro. Animals 11:1205. doi: 10.3390/ani11051205, PMID: 33922175 PMC8144957

[ref36] Trygg J. Wold S. (2002). Orthogonal projections to latent structures (O-PLS). J. Chemom. 16, 119–128. doi: 10.1002/cem.695

[ref37] Ussar S. Fujisaka S. Kahn C. R. (2016). Interactions between host genetics and gut microbiome in diabetes and metabolic syndrome. Mol. Metab. 5, 795–803. doi: 10.1016/j.molmet.2016.07.004, PMID: 27617202 PMC5004229

[ref38] Van Houcke J. Medina I. Maehre H. K. Cornet J. Cardinal M. Linssen J.. (2017). The effect of algae diets (*Skeletonema costatum* and *Rhodomonas baltica*) on the biochemical composition and sensory characteristics of Pacific cupped oysters (*Crassostrea gigas*) during land-based refinement. Food Res. Int. 100, 151–160. doi: 10.1016/j.foodres.2017.06.041, PMID: 28873674

[ref39] Wang Q. Garrity G. M. Tiedje J. M. Cole J. R. (2007). Naive Bayesian classifier for rapid assignment of rRNA sequences into the new bacterial taxonomy. Appl. Environ. Microbiol. 73, 5261–5267. doi: 10.1128/AEM.00062-07, PMID: 17586664 PMC1950982

[ref40] Wang W. Li C. Li F. Wang X. Zhang X. Liu T.. (2016). Effects of early feeding on the host rumen transcriptome and bacterial diversity in lambs. Sci. Rep. 6, 1–14. doi: 10.1038/srep32479, PMID: 27576848 PMC5006043

[ref41] Whitney T. Smith S. (2015). Substituting redberry juniper for oat hay in lamb feedlot diets: carcass characteristics, adipose tissue fatty acid composition, and sensory panel traits. Meat Sci. 104, 1–7. doi: 10.1016/j.meatsci.2015.01.010, PMID: 25678414

[ref42] Xiao J. Chen T. Alugongo G. M. Khan M. Z. Li T. Ma J.. (2021). Effect of the length of oat Hay on growth performance, health status, behavior parameters and rumen fermentation of Holstein female calves. Meta 11:890. doi: 10.3390/metabo11120890 PMC8703666 34940648

[ref43] Xu B. Xu W. Li J. Dai L. Xiong C. Tang X.. (2015). Metagenomic analysis of the Rhinopithecus bieti fecal microbiome reveals a broad diversity of bacterial and glycoside hydrolase profiles related to lignocellulose degradation. BMC Genom. 16, 174–111. doi: 10.1186/s12864-015-1378-7, PMID: 25887697 PMC4369366

[ref44] Yang J. Kurnia P. Henning S. M. Lee R. Huang J. Garcia M. C.. (2021). Effect of standardized grape powder consumption on the gut microbiome of healthy subjects: a pilot study. Nutrients 13:3965. doi: 10.3390/nu13113965, PMID: 34836220 PMC8619073

[ref45] Zhang Q. Xiao X. Li M. Yu M. Ping F. Zheng J.. (2017). Vildagliptin increases butyrate-producing bacteria in the gut of diabetic rats. PLoS One 12:e0184735. doi: 10.1371/journal.pone.0184735, PMID: 29036231 PMC5643055

[ref46] Zou Y. Zou X. Li X. Guo G. Ji P. Wang Y.. (2018). Substituting oat hay or maize silage for portion of alfalfa hay affects growth performance, ruminal fermentation, and nutrient digestibility of weaned calves. Asian Australas. J. Anim. Sci. 31, 369–378. doi: 10.5713/ajas.17.0210, PMID: 28728373 PMC5838342

