# Supplementary material for: Effects of oat (Avena sativa L.) hay diet supplementation on the intestinal microbiome and metabolome of Small-tail Han sheep
Source: Front Microbiol. 2022 Dec 16;13:1032622. doi: 10.3389/fmicb.2022.1032622 (PMC9801518; doi:10.3389/fmicb.2022.1032622)

Supplementary Material

**Supplementary Table 1．Ingredients and nutritional composition of the experimental diets (%, dry matter basis).**

| **Diet ingredient** | **Control**^2^ | **Oat**^2^ |
| --- | --- | --- |
| **Ingredients DM %** | | |
| [Maize straw](javascript:;) | 30.00 | -- |
| Oat hay | -- | 30.00 |
| Maize | 49.35 | 49.35 |
| Soybean meal | 17.15 | 17.15 |
| Ca(HCO_3_)_2_ | 0.70 | 0.70 |
| NaCl | 0.70 | 0.70 |
| NaHCO_3_ | 0.70 | 0.70 |
| Premix^1^ | 1.40 | 1.40 |
| Total | 100.00 | 100.00 |
| **Nutrient levels** |  |  |
| Metabolic energy (MJ/kg) | 9.08 | 10.17 |
| Crude protein (%) | 14.28 | 15.30 |
| Crude fat (%) | 2.22 | 2.85 |
| Calcium (%) | 0.81 | 0.85 |
| Phosphorus (%) | 0.53 | 0.49 |
| NDF^3^ (%) | 29.30 | 28.00 |
| ADF^4^ (%) | 12.60 | 12.76 |
| Dry matter (%) | 88.17 | 88.17 |

^1^ Premix provided the following per kg in basic diets:vitamin A, 280,000 IU;vitamin D 350,000 IU; vitamin E, 2,500 IU; D-biotine, 50 mg; β-carotene, 25 mg; Fe, 750 mg; Cu, 250 mg; Mn, 1400 mg; Zn, 3500 mg; Co, 30 mg; I, 55 mg; Se, 30 mg; ethoxyquin, 500 mg.

^2^ Oat:[experimental group](javascript:;);Control:[control group](javascript:;)

^3^ NDF: neutral detergent fibre

^4^ ADF：Acid detergent fiber

**Supplementary Table 2． Sample Tags Total table of distribution.**

| **Sample ID** | **Clean tags** | **Valid tags** | **Valid max Length** | **OTU counts** |
| --- | --- | --- | --- | --- |
| OatR-1 | 77759 | 71728 | 441 | 1841 |
| OatR-2 | 70740 | 65172 | 443 | 2128 |
| OatR-3 | 76619 | 70657 | 441 | 1926 |
| OatR-4 | 74850 | 67128 | 440 | 2622 |
| OatR-5 | 77051 | 72105 | 441 | 2029 |
| OatR-6 | 76848 | 68957 | 443 | 2180 |
| OatR-7 | 76457 | 67249 | 441 | 1556 |
| OatR-8 | 75397 | 68968 | 439 | 1727 |
| OatR-9 | 76802 | 68591 | 443 | 2747 |
| OatR-10 | 66518 | 57632 | 442 | 3235 |
| ControlR-1 | 74348 | 69138 | 442 | 1765 |
| ControlR-2 | 76209 | 67926 | 441 | 1636 |
| ControlR-3 | 77421 | 71474 | 440 | 1918 |
| ControlR-4 | 76527 | 69546 | 440 | 2404 |
| ControlR-5 | 74253 | 67643 | 439 | 2299 |
| ControlR-6 | 76940 | 69952 | 439 | 2734 |
| ControlR-7 | 74303 | 67932 | 440 | 2463 |
| ControlR-8 | 63822 | 54241 | 441 | 2929 |
| ControlR-9 | 76464 | 69823 | 442 | 2104 |
| OatC-1 | 77486 | 66440 | 441 | 3533 |
| OatC-2 | 74303 | 63100 | 442 | 3514 |
| OatC-3 | 73642 | 66441 | 457 | 1634 |
| OatC-4 | 57815 | 46861 | 455 | 3812 |
| OatC-5 | 72250 | 62246 | 441 | 3095 |
| OatC-6 | 75343 | 58626 | 442 | 3565 |
| OatC-7 | 77227 | 66243 | 441 | 3588 |
| OatC-8 | 73741 | 63529 | 443 | 2255 |
| OatC-9 | 75596 | 64592 | 440 | 3789 |
| OatC-10 | 74843 | 63182 | 440 | 3817 |
| ControlC-1 | 75469 | 75469 | 442 | 3120 |
| ControlC-2 | 70077 | 70077 | 441 | 2713 |
| ControlC-3 | 72880 | 72880 | 442 | 3093 |
| ControlC-4 | 76885 | 76885 | 442 | 3706 |
| ControlC-5 | 74025 | 74025 | 441 | 2459 |
| ControlC-6 | 75071 | 75071 | 441 | 3119 |
| ControlC-7 | 74775 | 74775 | 441 | 3843 |
| ControlC-8 | 74800 | 74800 | 439 | 3568 |
| ControlC-9 | 72812 | 72812 | 441 | 3642 |
| ControlC-10 | 72370 | 72370 | 441 | 4071 |

**Supplementary Table 3． Model parameters of rumen and colon samples were compared in pairs under three feeding conditions.**

| Organ | Group | Type | PRE | ORT | N | R2X(cum) | R2Y(cum) | Q2(cum) |
| --- | --- | --- | --- | --- | --- | --- | --- | --- |
| Rumen | OatR/ControlR | PCA | 5 | 0 | 20 | 0.554 |  |  |
|  |  | OPLS | 1 | 2 | 20 | 0.497 | 0.95 | 0.254 |
| Colon | OatC/ControlC | PCA | 4 | 0 | 20 | 0.510 |  |  |
|  |  | OPLS | 1 | 3 | 20 | 0.680 | 0.977 | 0.600 |

Type: established multivariate statistical analysis model;

PRE: represents the number of principal components in modeling;

ORT: represents the number of orthogonal components in modeling;

N:represents the number of samples during modeling;

R2X(CUM) : represents the cumulative interpretation rate of the X-axis model in multivariate statistical analysis modeling, and CUM represents the cumulative results of several principal components.

R2Y(CUM) : represents the cumulative interpretation rate of the model in the Y-axis direction;

Q2(CUM) : Represents the cumulative prediction rate of the model.

**Supplementary Figure 1. Venn diagram of OTUs in the colon microbiota.** OatC = Colon samples from oat group, ControlC=Colon samples from control group.


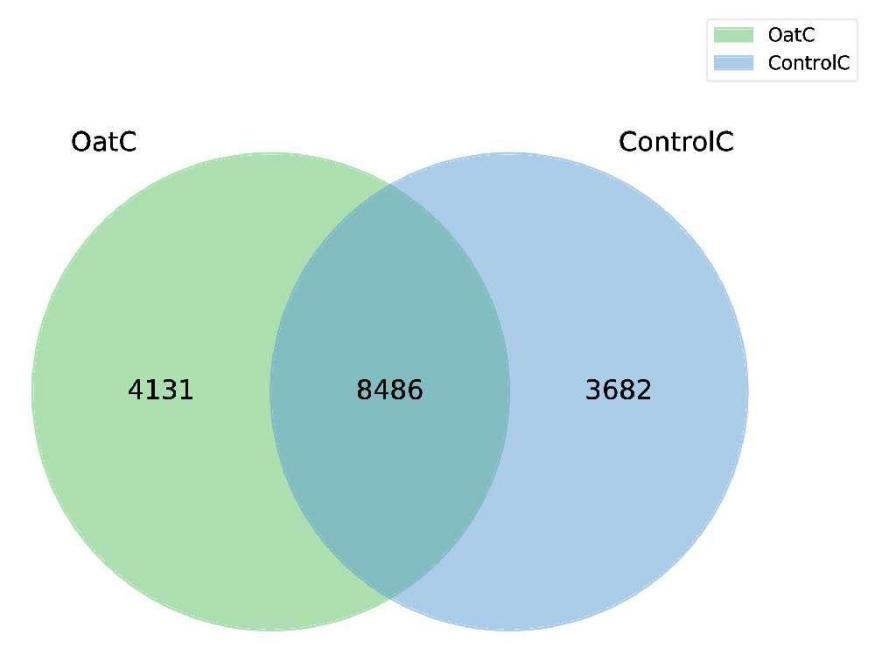


**Supplementary Figure 2. OTU diversity index dilution curve.**


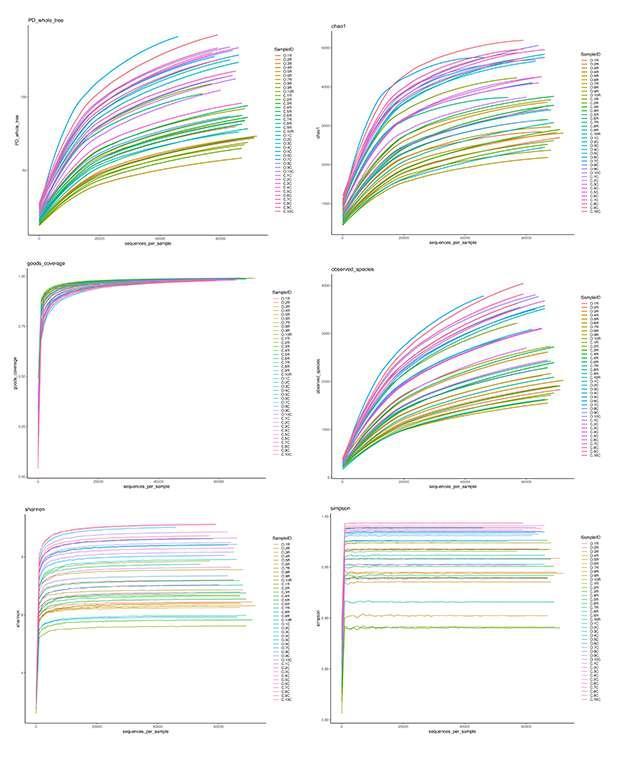


**Supplementary Figure 3. Top10 boxplot of different species abundance at phylum level.**


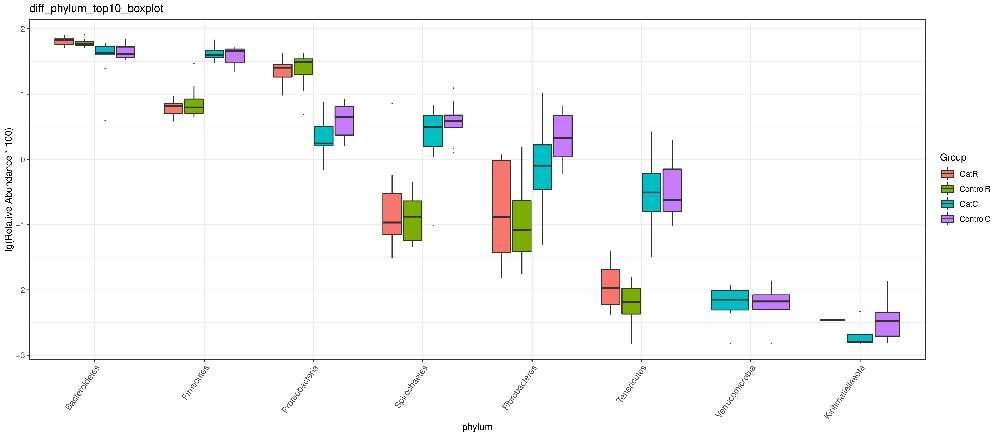


**Supplementary Figure 4. Top10 boxplot of different species abundance at family level.**


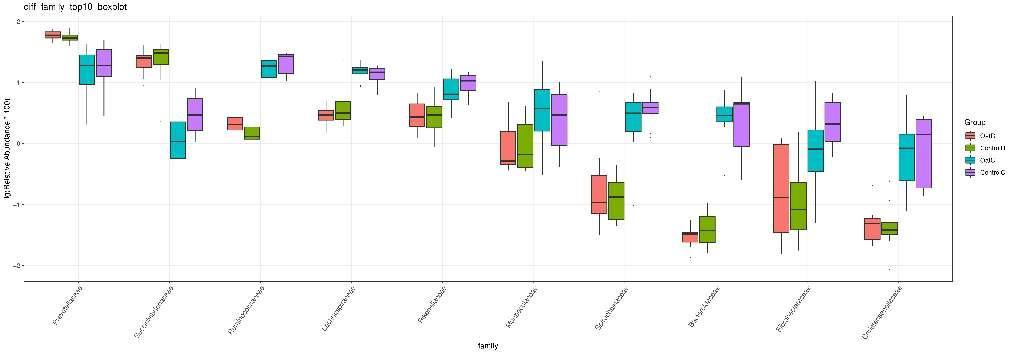


**Supplementary Figure 5. Top10 boxplot of different species abundance at genus level in the rumen.**


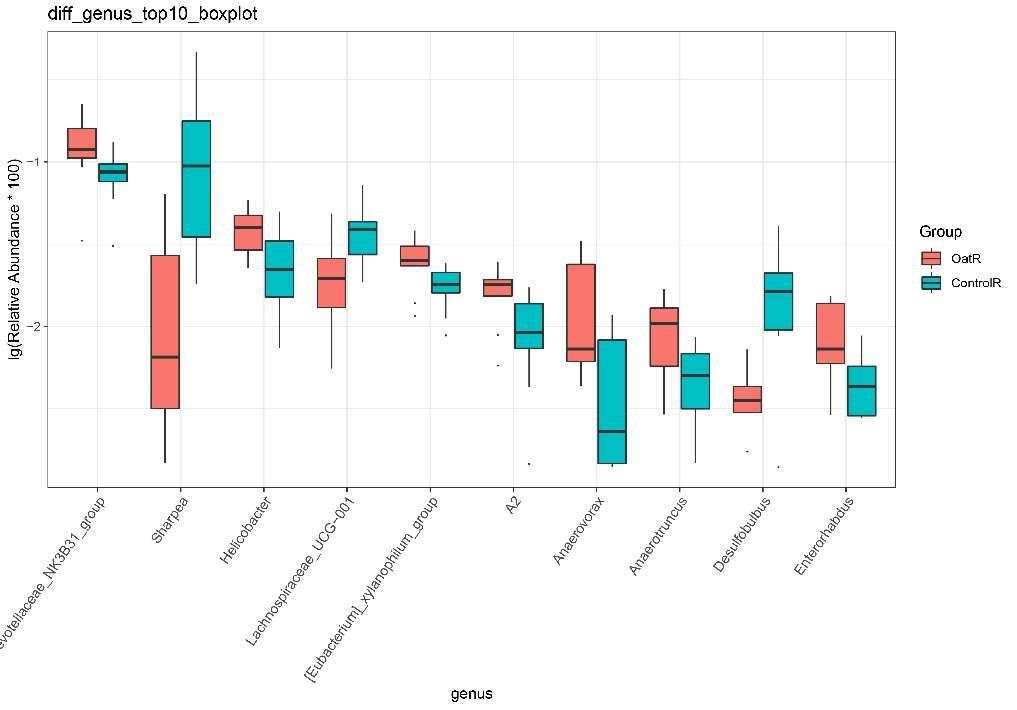


**Supplementary Figure 6. The second-level KEGG difference results were clustered into heatmap.** OatR=Rumen samples from oat group, ControlR=Rumen samples from control group.


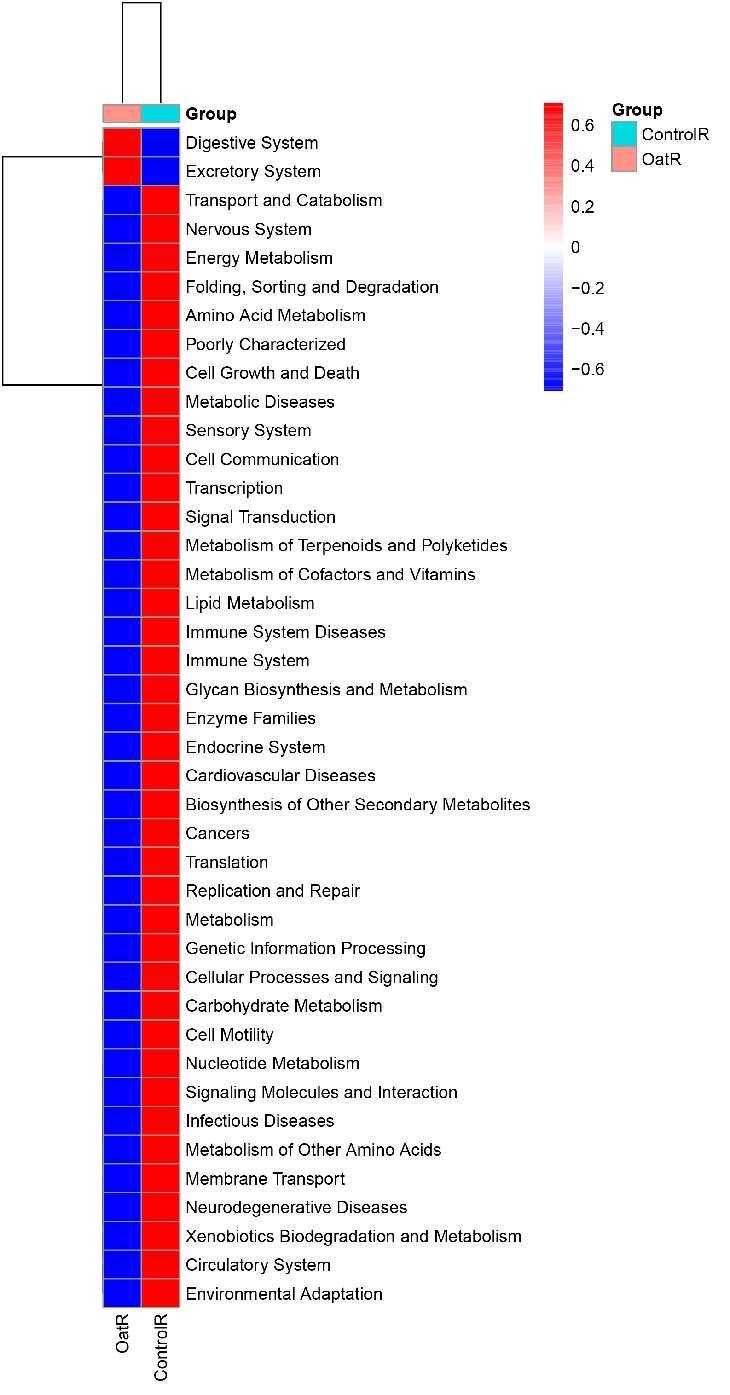


**Supplementary Figure 7. Plot of PCA (A) and OPLS-DA (B) score, and OPLS-DA permutation (C), as well as volcano plot (D) in the rumen group.**


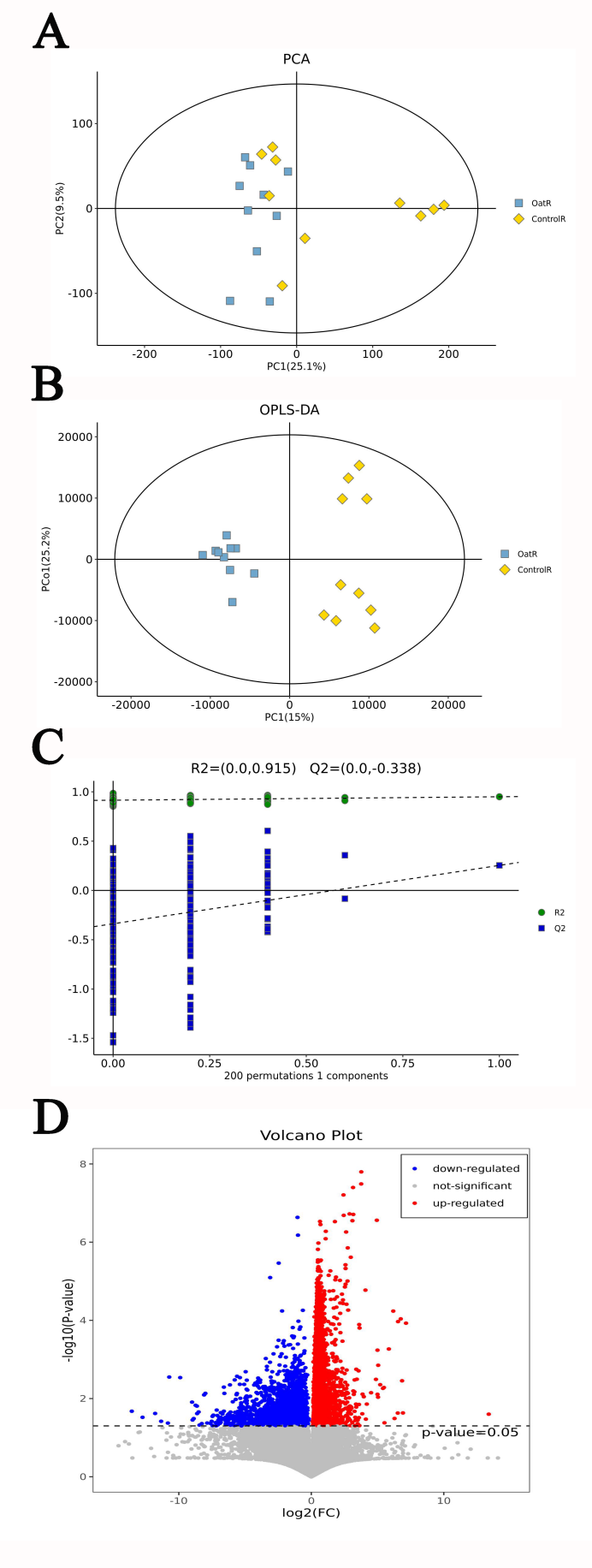


**Supplementary Figure 8. The spearman correlation network between ruminal microbiome and metabolome in OatR vs ControlR. * *P* < 0.05; ** *P* < 0.01.**


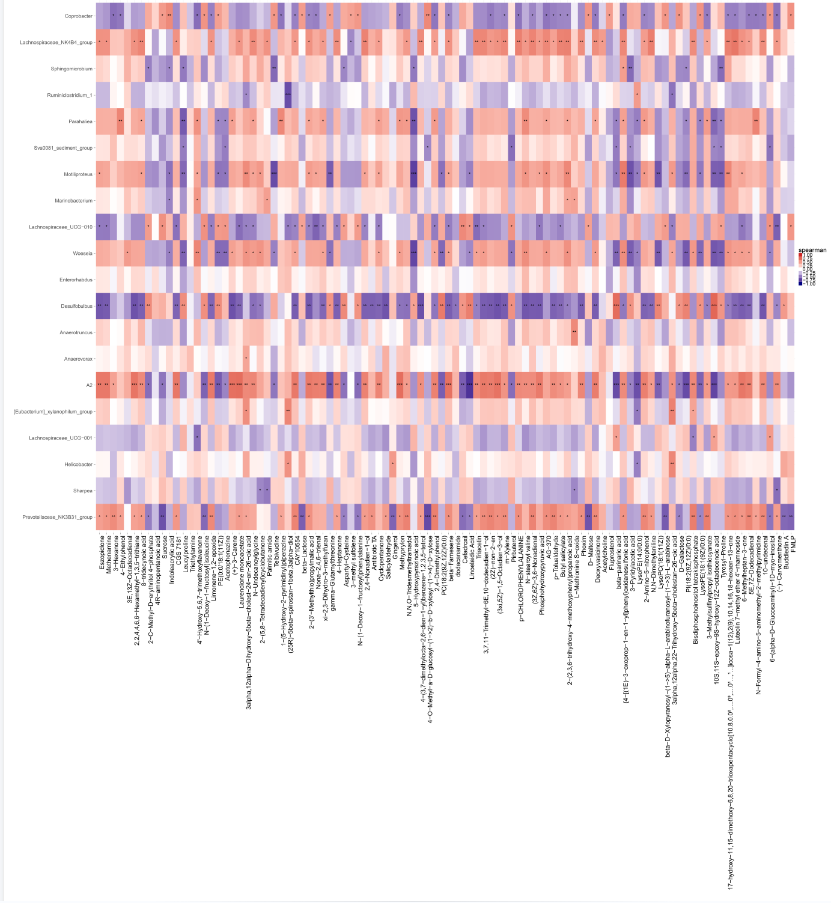

Supplement: Supplementary file 1 [file Data_Sheet_1.docx]
